# Poly[di-μ_9_-citrato-cobalt(II)tetra­sodium]

**DOI:** 10.1107/S1600536812017606

**Published:** 2012-04-25

**Authors:** Zhen Liu, Ruijing Tian, Rui Mao, Xueyin Cao, Fuxiang Wang

**Affiliations:** a Department of Materials and Chemical Engineering, Ministry of Education Key Laboratory of Advanced Materials of Tropical Island Resources, Hainan University, Haikou 570228, Hainan Province, People’s Republic of China

## Abstract

The title compound, [CoNa_4_(C_6_H_5_O_7_)_2_]_*n*_, was obtained under hydro­thermal conditions as a minor product. The Co^2+^ cation is located on a crystallographic inversion center and is coordinated by six O atoms from two different citrate units, forming a [Co(C_6_H_5_O_7_)_2_]^4−^ building unit with Co—O bond lengths between 2.0578 (17) and 2.0813 (16) Å. The structure features two crystallographically independent Na^+^ ions. The first Na^+^ cation is five-coordinated by O atoms of five carboxylate groups from four different citrate anions. The second Na^+^ cation is surrounded by six O atoms of five carboxylate groups from five different citrate anions. The carboxylate groups of the citrate are completely depronona­ted, the hydroxyl group, however, is not. It is coordinated to the Co^2+^ cation, and through an O—H⋯O hydrogen bond connected to a neighboring [Co(C_6_H_5_O_7_)_2_]^4−^ building unit. The coordination modes of the carboxyl­ate O atoms vary, with one O atom being coordinated to three different Na^+^ cations, three are bridging O atoms bound to two Na^+^ cations and two are connected to a Co^2+^ cation and a Na^+^ cation, respectively. Through these inter­connections, the basic [Co(C_6_H_5_O_7_)_2_]^4−^ building units are linked with each other through coordination of their carboxyl­ate groups to the Na^+^ cations, forming a three-dimensional framework.

## Related literature

For potential applications of coordination polymers in drug delivery, shape-selective sorption/separation and catalysis, see: Chen & Tong (2007 ▶); Zeng *et al.* (2009 ▶). Their structures vary from one-dimensional to three-dimensional architectures, see: Du & Bu *et al.* (2009 ▶); Qiu & Zhu (2009 ▶). For a compound containing the [Co(C_6_H_5_O_7_)_2_]^4−^ subunit, see: Matzapetakis *et al.* (2000 ▶); for coordination polymers involving Na^+^ cations, see: Pan *et al.* (2011 ▶).
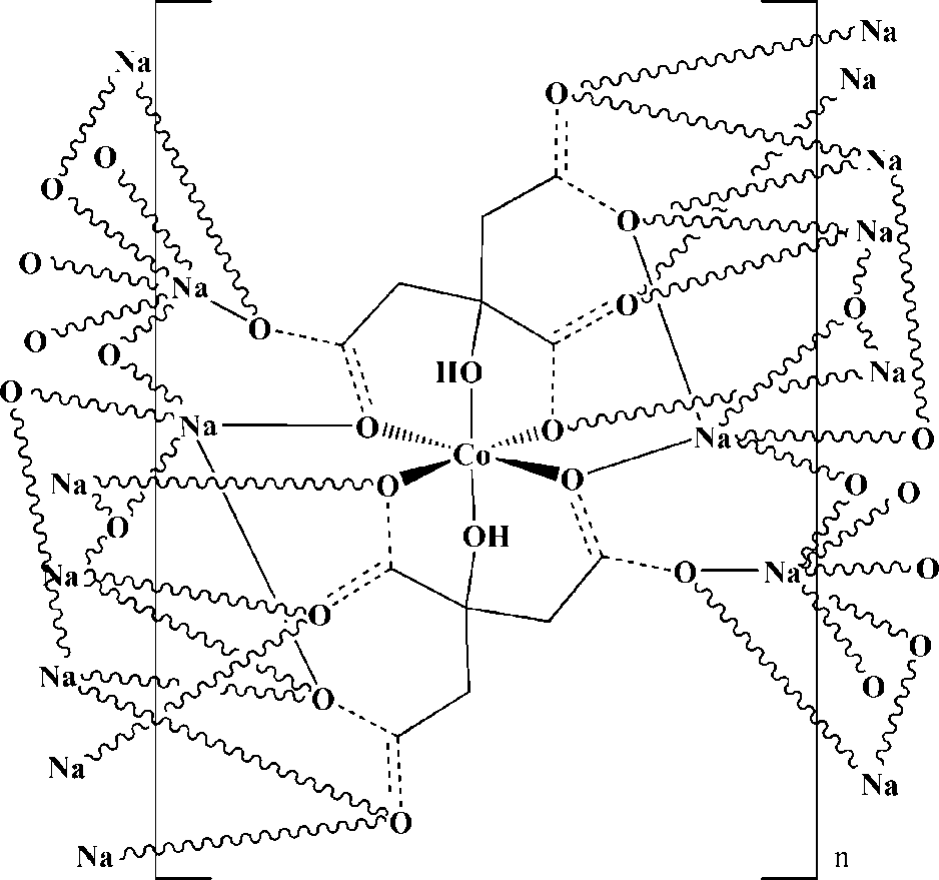


## Experimental

### 

#### Crystal data

[CoNa_4_(C_6_H_5_O_7_)_2_]
*M* *_r_* = 529.09
Monoclinic, 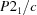

*a* = 7.9792 (16) Å
*b* = 12.516 (3) Å
*c* = 8.7110 (17) Å
β = 113.84 (3)°
*V* = 795.7 (3) Å^3^
*Z* = 2
Mo *K*α radiation
μ = 1.28 mm^−1^
*T* = 293 K
0.25 × 0.15 × 0.15 mm

#### Data collection

Rigaku R-AXIS RAPID-S diffractometer
Absorption correction: multi-scan (*CrystalClear*; Rigaku/MSC, 2002 ▶) *T*_min_ = 0.795, *T*_max_ = 0.826
8179 measured reflections
1820 independent reflections
1493 reflections with *I* > 2σ(*I*)
*R*_int_ = 0.055

#### Refinement

*R*[*F* ^2^ > 2σ(*F* ^2^)] = 0.041
*wR*(*F* ^2^) = 0.072
*S* = 1.12
1820 reflections
142 parameters
H-atom parameters constrained
Δρ_max_ = 0.29 e Å^−3^
Δρ_min_ = −0.31 e Å^−3^

### 

Data collection: *RAPID-AUTO* (Rigaku, 1998 ▶); cell refinement: *RAPID-AUTO*; data reduction: *CrystalStructure* (Rigaku/MSC, 2002 ▶); program(s) used to solve structure: *SHELXS97* (Sheldrick, 2008 ▶); program(s) used to refine structure: *SHELXL97* (Sheldrick, 2008 ▶); molecular graphics: *SHELXTL* (Sheldrick, 2008 ▶); software used to prepare material for publication: *SHELXTL*.

## Supplementary Material

Crystal structure: contains datablock(s) I, global. DOI: 10.1107/S1600536812017606/zl2468sup1.cif

Structure factors: contains datablock(s) I. DOI: 10.1107/S1600536812017606/zl2468Isup2.hkl

Additional supplementary materials:  crystallographic information; 3D view; checkCIF report

## Figures and Tables

**Table 1:** Hydrogen-bond geometry (Å, °)

*D*—H⋯*A*	*D*—H	H⋯*A*	*D*⋯*A*	*D*—H⋯*A*
O1—H1⋯O4^i^	0.85	1.79	2.640 (3)	174

Symmetry code: (i) 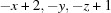.

**Fig. 1.**
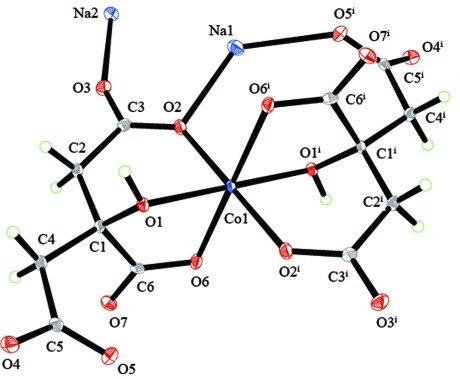
A view of the asymmetric unit of complex. Ellipsoids are drawn at the 30% probability level. [Symmetry code: (i) 2-x, -y, 2-z.]

**Fig. 2.**
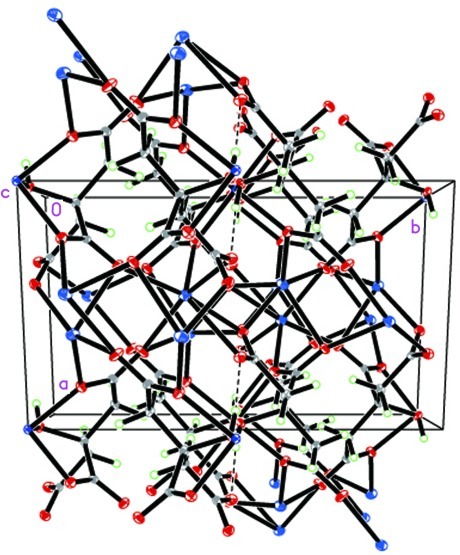
A view of the packing along the a axis. Dashed lines indicate hydrogen bonds.

**Fig. 3.**
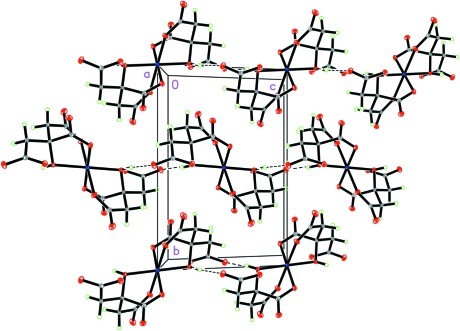
A view of the arrangement of the [Co(C6H5O7)2]4- building units along the bc palne. Dashed lines indicate hydrogen bonds. Sodium atoms are omitted in this view for clarity.

**Fig. 4.**
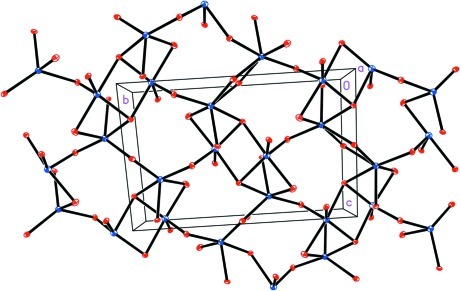
A view of Na—O sheet along the bc plane. Co, C and H atoms are omitted in this view for clarity.

## supplementary crystallographic information

### Comment

The design and synthesis of coordination polymers have attracted increasing attention in recent years because of their potential applications in drug delivery, shape-selective sorption/separation, and catalysis (Chen *et al.*, 2007, Zeng *et al.*, 2009). Architectures of coordination polymers described vary between one-dimensional and three-dimensional (Qiu *et al.*, 2009, Du *et al.*, 2009). Citric acid and its anions have been widely used as ligands for the construction of coordination polymers, because of their versatility and ability to bind metals with diverse connection modes. In this paper, we present a new three-dimensional coordination polymer [Na_4_Co(C_6_H_5_O_7_)_2_]_n_ with citric acid as the ligand.

As shown in Fig. 1, the asymmetric unit of the crystal structure of the title compound consists of half a Co^2+^ center, two Na^+^ cations, and a citrate anion. The Co^2+^ center, located on a crystallographic inversion center, is coordinated by six O atoms from two different citrate units with the Co—O bond distances in the range of 2.0578 (17) to 2.0812 (16) Å, resulting in a slightly distorted octahedral coordination geometry. For the citrate unit, all the carboxylic acid groups are completely deprononated, however the hydroxyl group is not. Three O atoms of each citrate unit are bonded to the Co^2+^ center, one of which is the hydroxy O atom of the citric acid, the other two are from two different carboxylate groups of the citrate ligands. In such a way, two citrate anions and one Co^2+^ cation form a [Co(C_6_H_5_O_7_)_2_]^4-^ building unit such as described previously by Matzapetakis *et al.* (2000). The citrate anions also coordinate to the Na^+^ cations. Oxygen atom O1 of the hydroxyl group is connected only to the Co cation. Carboxylate oxygen atom O5 is linked to three different Na cations. The other five O atoms are bridging O atoms bound to two Na cations (O3, O4, and O7 atoms) or a Co cation and a Na cation (O2 and O6 atoms), respectively. The two Na^+^ cations display different coordination modes by the O atoms. The Na1^+^ cation is five-coordinated by five O atoms of five carboxylic acid groups from four different citrate units. The Na2^+^ cation, on the other hand, is surrounded by six O atoms of five carboxylic acid groups from five different citrate units. The Na—O bond distances are in the range of 2.286 (2)–2.562 (2) Å, which is in the usual range expected for such a compound (see for example: Pan *et al.*, 2011). In this way, the [Co(C_6_H_5_O_7_)_2_]^4-^ building units are linked with each other through coordination of their carboxylate groups to the Na^+^ cations to form a three-dimensional framework (see Fig. 2).

As shown in Fig. 3, the [Co(C_6_H_5_O_7_)_2_]^4-^ building units are arranged in layers with the Co^2+^ centers located within the *bc* plane (Co atoms are placed on crystallographic inversion centers at the corners of the unit cell and at the center of the *bc* plane of the unit cell). Neighboring [Co(C_6_H_5_O_7_)_2_]^4-^ building units are further linked along the *c* axis to form a one-dimesional chain, and by the O—H···O hydrogen bonds formed by the hydroxyl group and the O4 atom of a neighboring building unit (symmetry code: -*x*+2, -*y*, -*z*+1). The Na^+^ cations are located between adjacent planes of the cobalt building units, and are coordinated by the carboxylate groups of citrate ligands. In such a way a sheet dominated by Na—O ionic interactions is formed, as shown in Fig. 4. The sheets are arranged parallel to those of the Co(C_6_H_5_O_7_)_2_]^4-^ building units perpendicular to the *a* axis, and are supported by the [Co(C_6_H_5_O_7_)_2_]^4-^ building units to construct the three-dimensional coordination polymer.

### Experimental

In a typical synthesis, a mixture of Co(OAc)_2_.4H_2_O (0.08 g), benzene-1,4-dicarboxylic acid (0.042 g), NaOH (0.012 g), citric acid (0.096 g), H_2_O (3 ml) and ethanol (7 ml) was added to a 20 ml Teflon-lined stainless steel autoclave and heated at 433 K (160 °C) for 3 days. Yellow block shaped crystals were obtained. yield: ~15% (based on NaOH).

### Refinement

All H atoms were positioned geometrically (C—H = 0.97 Å and O—H = 0.85 Å) and allowed to ride on their parent atoms, with *U*_iso_(H) = 1.2*U*_eq_(parent atom).

### Crystal data

**Table:**

[CoNa_4_(C_6_H_5_O_7_)_2_]	*F*(000) = 530
*M**_r_* = 529.09	*D*_x_ = 2.208 Mg m^−^^3^
Monoclinic, *P*2_1_/*c*	Mo *K*α radiation, λ = 0.71073 Å
Hall symbol: -P 2ybc	Cell parameters from 7365 reflections
*a* = 7.9792 (16) Å	θ = 3.0–27.5°
*b* = 12.516 (3) Å	µ = 1.28 mm^−^^1^
*c* = 8.7110 (17) Å	*T* = 293 K
β = 113.84 (3)°	Block, yellow
*V* = 795.7 (3) Å^3^	0.25 × 0.15 × 0.15 mm
*Z* = 2	

### Data collection

**Table:**

Rigaku R-AXIS RAPID-S diffractometer	1820 independent reflections
Radiation source: fine-focus sealed tube	1493 reflections with *I* > 2σ(*I*)
Graphite monochromator	*R*_int_ = 0.055
ω scans	θ_max_ = 27.5°, θ_min_ = 3.0°
Absorption correction: multi-scan (*CrystalClear*; Rigaku/MSC, 2002)	*h* = −10→10
*T*_min_ = 0.795, *T*_max_ = 0.826	*k* = −15→16
8179 measured reflections	*l* = −11→11

### Refinement

**Table:**

Refinement on *F*^2^	Primary atom site location: structure-invariant direct methods
Least-squares matrix: full	Secondary atom site location: difference Fourier map
*R*[*F*^2^ > 2σ(*F*^2^)] = 0.041	Hydrogen site location: inferred from neighbouring sites
*wR*(*F*^2^) = 0.072	H-atom parameters constrained
*S* = 1.12	*w* = 1/[σ^2^(*F*_o_^2^) + (0.0253*P*)^2^ + 0.3552*P*] where *P* = (*F*_o_^2^ + 2*F*_c_^2^)/3
1820 reflections	(Δ/σ)_max_ < 0.001
142 parameters	Δρ_max_ = 0.29 e Å^−^^3^
0 restraints	Δρ_min_ = −0.31 e Å^−^^3^

### Special details

**Table:**

Geometry. All e.s.d.'s (except the e.s.d. in the dihedral angle between two l.s. planes) are estimated using the full covariance matrix. The cell e.s.d.'s are taken into account individually in the estimation of e.s.d.'s in distances, angles and torsion angles; correlations between e.s.d.'s in cell parameters are only used when they are defined by crystal symmetry. An approximate (isotropic) treatment of cell e.s.d.'s is used for estimating e.s.d.'s involving l.s. planes.
Refinement. Refinement of *F*^2^ against ALL reflections. The weighted *R*-factor *wR* and goodness of fit *S* are based on *F*^2^, conventional *R*-factors *R* are based on *F*, with *F* set to zero for negative *F*^2^. The threshold expression of *F*^2^ > σ(*F*^2^) is used only for calculating *R*-factors(gt) *etc*. and is not relevant to the choice of reflections for refinement. *R*-factors based on *F*^2^ are statistically about twice as large as those based on *F*, and *R*- factors based on ALL data will be even larger.

### Fractional atomic coordinates and isotropic or equivalent isotropic displacement parameters (Å^2^)

**Table:**

	*x*	*y*	*z*	*U*_iso_*/*U*_eq_	
Co1	1.0000	0.0000	1.0000	0.01112 (13)	
Na1	0.60461 (14)	0.11777 (8)	1.03513 (13)	0.0233 (3)	
Na2	0.44536 (14)	0.37904 (8)	0.83883 (13)	0.0219 (3)	
O1	1.0085 (2)	0.01241 (12)	0.7650 (2)	0.0125 (4)	
H1	0.9089	0.0001	0.6807	0.015*	
O2	0.8226 (2)	0.12772 (13)	0.9279 (2)	0.0177 (4)	
O3	0.6796 (2)	0.27068 (15)	0.7861 (2)	0.0232 (5)	
O4	1.3026 (2)	0.01125 (14)	0.5009 (2)	0.0195 (4)	
O5	1.4004 (2)	0.01690 (13)	0.7789 (2)	0.0186 (4)	
O6	1.2231 (2)	0.10114 (14)	1.0518 (2)	0.0172 (4)	
O7	1.3273 (2)	0.22348 (13)	0.9263 (2)	0.0187 (4)	
C1	1.0686 (3)	0.12024 (19)	0.7524 (3)	0.0120 (5)	
C2	0.9109 (3)	0.19974 (19)	0.7122 (3)	0.0138 (5)	
H2A	0.9616	0.2709	0.7193	0.017*	
H2B	0.8274	0.1886	0.5961	0.017*	
C3	0.7974 (3)	0.1990 (2)	0.8171 (3)	0.0143 (5)	
C4	1.1394 (3)	0.12288 (19)	0.6139 (3)	0.0144 (5)	
H4A	1.0377	0.1087	0.5075	0.017*	
H4B	1.1833	0.1944	0.6082	0.017*	
C5	1.2912 (3)	0.0446 (2)	0.6347 (3)	0.0142 (5)	
C6	1.2209 (3)	0.1507 (2)	0.9229 (3)	0.0132 (5)	

### Atomic displacement parameters (Å^2^)

**Table:**

	*U*^11^	*U*^22^	*U*^33^	*U*^12^	*U*^13^	*U*^23^
Co1	0.0127 (2)	0.0116 (2)	0.0100 (2)	0.0002 (2)	0.00553 (18)	0.0019 (2)
Na1	0.0213 (6)	0.0273 (6)	0.0241 (6)	−0.0021 (5)	0.0121 (5)	−0.0007 (5)
Na2	0.0197 (5)	0.0251 (6)	0.0217 (6)	−0.0023 (5)	0.0092 (5)	0.0015 (5)
O1	0.0139 (8)	0.0118 (9)	0.0109 (8)	−0.0036 (7)	0.0041 (7)	−0.0007 (7)
O2	0.0205 (10)	0.0181 (10)	0.0184 (10)	0.0034 (8)	0.0121 (8)	0.0051 (8)
O3	0.0212 (11)	0.0269 (11)	0.0246 (11)	0.0126 (8)	0.0126 (9)	0.0074 (9)
O4	0.0186 (10)	0.0256 (10)	0.0163 (9)	0.0018 (8)	0.0092 (8)	−0.0050 (8)
O5	0.0190 (9)	0.0187 (10)	0.0152 (9)	0.0030 (8)	0.0039 (8)	0.0003 (8)
O6	0.0176 (9)	0.0199 (10)	0.0113 (9)	−0.0018 (8)	0.0028 (7)	0.0032 (8)
O7	0.0179 (10)	0.0176 (9)	0.0219 (10)	−0.0051 (8)	0.0096 (8)	−0.0022 (8)
C1	0.0144 (12)	0.0112 (12)	0.0118 (12)	0.0005 (10)	0.0069 (10)	0.0026 (10)
C2	0.0167 (13)	0.0145 (12)	0.0111 (12)	0.0030 (10)	0.0066 (10)	0.0015 (11)
C3	0.0137 (13)	0.0150 (13)	0.0128 (13)	0.0000 (10)	0.0038 (10)	−0.0023 (11)
C4	0.0167 (13)	0.0151 (13)	0.0125 (13)	0.0032 (10)	0.0072 (11)	0.0015 (11)
C5	0.0139 (13)	0.0135 (12)	0.0160 (14)	−0.0039 (10)	0.0068 (11)	−0.0002 (11)
C6	0.0112 (12)	0.0141 (12)	0.0164 (13)	0.0042 (10)	0.0077 (11)	−0.0021 (11)

### Geometric parameters (Å, º)

**Table:**

Co1—O2^i^	2.0578 (17)	O4—C5	1.275 (3)
Co1—O2	2.0578 (17)	O4—Na2^viii^	2.542 (2)
Co1—O6	2.0800 (17)	O4—Na2^ix^	2.545 (2)
Co1—O6^i^	2.0800 (17)	O5—C5	1.254 (3)
Co1—O1	2.0813 (16)	O5—Na1^i^	2.349 (2)
Co1—O1^i^	2.0813 (16)	O5—Na1^x^	2.508 (2)
Na1—O2	2.286 (2)	O5—Na2^viii^	2.562 (2)
Na1—O5^i^	2.349 (2)	O6—C6	1.276 (3)
Na1—O7^ii^	2.418 (2)	O6—Na2^xi^	2.424 (2)
Na1—O3^iii^	2.455 (2)	O7—C6	1.238 (3)
Na1—O5^ii^	2.508 (2)	O7—Na2^x^	2.416 (2)
Na2—O7^ii^	2.416 (2)	O7—Na1^x^	2.418 (2)
Na2—O6^iv^	2.424 (2)	C1—C4	1.526 (3)
Na2—O3	2.498 (2)	C1—C2	1.530 (3)
Na2—O4^v^	2.542 (2)	C1—C6	1.539 (3)
Na2—O4^vi^	2.545 (2)	C2—C3	1.525 (3)
Na2—O5^v^	2.562 (2)	C2—H2A	0.9700
O1—C1	1.451 (3)	C2—H2B	0.9700
O1—H1	0.8501	C4—C5	1.510 (3)
O2—C3	1.270 (3)	C4—H4A	0.9700
O3—C3	1.247 (3)	C4—H4B	0.9700
O3—Na1^vii^	2.455 (2)		
			
O2^i^—Co1—O2	180.000 (1)	C3—O3—Na1^vii^	119.56 (16)
O2^i^—Co1—O6	89.06 (7)	C3—O3—Na2	154.82 (17)
O2—Co1—O6	90.94 (7)	Na1^vii^—O3—Na2	85.63 (7)
O2^i^—Co1—O6^i^	90.94 (7)	C5—O4—Na2^viii^	92.53 (15)
O2—Co1—O6^i^	89.06 (7)	C5—O4—Na2^ix^	122.91 (16)
O6—Co1—O6^i^	180.0	Na2^viii^—O4—Na2^ix^	102.91 (7)
O2^i^—Co1—O1	93.79 (7)	C5—O5—Na1^i^	133.69 (16)
O2—Co1—O1	86.21 (7)	C5—O5—Na1^x^	133.68 (16)
O6—Co1—O1	78.61 (7)	Na1^i^—O5—Na1^x^	86.18 (7)
O6^i^—Co1—O1	101.39 (7)	C5—O5—Na2^viii^	92.13 (15)
O2^i^—Co1—O1^i^	86.21 (7)	Na1^i^—O5—Na2^viii^	86.42 (6)
O2—Co1—O1^i^	93.79 (7)	Na1^x^—O5—Na2^viii^	116.79 (8)
O6—Co1—O1^i^	101.39 (7)	C6—O6—Co1	113.50 (16)
O6^i^—Co1—O1^i^	78.61 (7)	C6—O6—Na2^xi^	127.01 (16)
O1—Co1—O1^i^	180.000 (1)	Co1—O6—Na2^xi^	119.41 (8)
O2—Na1—O5^i^	123.27 (8)	C6—O7—Na2^x^	159.49 (17)
O2—Na1—O7^ii^	122.46 (7)	C6—O7—Na1^x^	96.72 (15)
O5^i^—Na1—O7^ii^	113.26 (7)	Na2^x^—O7—Na1^x^	98.80 (7)
O2—Na1—O3^iii^	112.38 (8)	O1—C1—C4	108.62 (19)
O5^i^—Na1—O3^iii^	81.95 (7)	O1—C1—C2	110.85 (19)
O7^ii^—Na1—O3^iii^	83.86 (7)	C4—C1—C2	109.76 (19)
O2—Na1—O5^ii^	89.53 (7)	O1—C1—C6	108.27 (19)
O5^i^—Na1—O5^ii^	93.82 (7)	C4—C1—C6	110.9 (2)
O7^ii^—Na1—O5^ii^	76.36 (7)	C2—C1—C6	108.40 (19)
O3^iii^—Na1—O5^ii^	156.27 (7)	C3—C2—C1	119.5 (2)
O7^ii^—Na2—O6^iv^	101.07 (7)	C3—C2—H2A	107.5
O7^ii^—Na2—O3	92.10 (7)	C1—C2—H2A	107.5
O6^iv^—Na2—O3	98.95 (7)	C3—C2—H2B	107.5
O7^ii^—Na2—O4^v^	132.38 (7)	C1—C2—H2B	107.5
O6^iv^—Na2—O4^v^	125.90 (7)	H2A—C2—H2B	107.0
O3—Na2—O4^v^	88.26 (7)	O3—C3—O2	123.0 (2)
O7^ii^—Na2—O4^vi^	86.60 (7)	O3—C3—C2	116.4 (2)
O6^iv^—Na2—O4^vi^	102.26 (7)	O2—C3—C2	120.6 (2)
O3—Na2—O4^vi^	158.61 (8)	C5—C4—C1	115.2 (2)
O4^v^—Na2—O4^vi^	77.09 (7)	C5—C4—H4A	108.5
O7^ii^—Na2—O5^v^	168.65 (7)	C1—C4—H4A	108.5
O6^iv^—Na2—O5^v^	77.74 (7)	C5—C4—H4B	108.5
O3—Na2—O5^v^	77.04 (7)	C1—C4—H4B	108.5
O4^v^—Na2—O5^v^	51.67 (6)	H4A—C4—H4B	107.5
O4^vi^—Na2—O5^v^	104.71 (7)	O5—C5—O4	123.2 (2)
C1—O1—Co1	106.67 (13)	O5—C5—C4	119.9 (2)
C1—O1—H1	109.0	O4—C5—C4	116.9 (2)
Co1—O1—H1	116.4	O7—C6—O6	124.8 (2)
C3—O2—Co1	131.01 (16)	O7—C6—C1	118.2 (2)
C3—O2—Na1	115.84 (16)	O6—C6—C1	117.0 (2)
Co1—O2—Na1	112.10 (8)		

Symmetry codes: (i) −*x*+2, −*y*, −*z*+2; (ii) *x*−1, *y*, *z*; (iii) *x*, −*y*+1/2, *z*+1/2; (iv) *x*−1, −*y*+1/2, *z*−1/2; (v) −*x*+2, *y*+1/2, −*z*+3/2; (vi) *x*−1, −*y*+1/2, *z*+1/2; (vii) *x*, −*y*+1/2, *z*−1/2; (viii) −*x*+2, *y*−1/2, −*z*+3/2; (ix) *x*+1, −*y*+1/2, *z*−1/2; (x) *x*+1, *y*, *z*; (xi) *x*+1, −*y*+1/2, *z*+1/2.

### Hydrogen-bond geometry (Å, º)

**Table:**

*D*—H···*A*	*D*—H	H···*A*	*D*···*A*	*D*—H···*A*
O1—H1···O4^xii^	0.85	1.79	2.640 (3)	174

Symmetry code: (xii) −*x*+2, −*y*, −*z*+1.
